# CRISPR/Cas9-Mediated CtBP1 Gene Editing Enhances Chemosensitivity and Inhibits Metastatic Potential in Esophageal Squamous Cell Carcinoma Cells

**DOI:** 10.3390/ijms241814030

**Published:** 2023-09-13

**Authors:** Javed Akhtar, Muhammad Imran, Guanyu Wang

**Affiliations:** 1 Futian Biomedical Innovation R&D Center, The Chinese University of Hong Kong, Shenzhen 518172, China; javedakhtar@cuhk.edu.cn; 2 Biomedical Science and Engineering, School of Medicine, The Chinese University of Hong Kong, Shenzhen 518172, China; 3 Ciechanover Institute of Precision and Regenerative Medicine, School of Medicine, The Chinese University of Hong Kong, Shenzhen 518172, China; 4 Center for Endocrinology and Metabolic Diseases, Second Affiliated Hospital, The Chinese University of Hong Kong, Shenzhen 518172, China; 5 Department of Computer Science & IT, Institute of Southern Punjab, Multan 60800, Pakistan; stunningimran50@gmail.com

**Keywords:** esophageal squamous cell carcinoma (ESCC), paclitaxel resistance, C-Terminal Binding Protein 1 (CtBP1), CRISPR/Cas9 genome editing

## Abstract

Innovative therapeutic strategies for esophageal squamous cell carcinoma (ESCC) are urgently required due to the limited effectiveness of standard chemotherapies. C-Terminal Binding Protein 1 (CtBP1) has been implicated in various cancers, including ESCC. However, the precise expression patterns and functional roles of CtBP1 in ESCC remain inadequately characterized. In this study, we aimed to investigate CtBP1 expression and its role in the resistance of ESCC to paclitaxel, an effective chemotherapeutic agent. Western blotting and immunofluorescence were applied to assess CtBP1 expression in the TE-1 and KYSE-50 cell lines. We observed the marked expression of CtBP1, which was associated with enhanced proliferation, invasion, and metastasis in these cell lines. Further, we successfully generated paclitaxel resistant ESCC cell lines and conducted cell viability assays. We employed the CRISPR/Cas9 genome editing system to disable the CtBP1 gene in ESCC cell lines. Through the analysis of the drug dose–response curve, we assessed the sensitivity of these cell lines in different treatment groups. Remarkably, CtBP1-disabled cell lines displayed not only improved sensitivity but also a remarkable inhibition of proliferation, invasion, and metastasis. This demonstrates that CtBP1 may promote ESCC cell malignancy and confer paclitaxel resistance. In summary, our study opens a promising avenue for targeted therapies, revealing the potential of CtBP1 inhibition to enhance the effectiveness of paclitaxel treatment for the personalized management of ESCC.

## 1. Introduction

Esophageal cancer (EC) ranks the eighth most diagnosed cancer and the sixth most prevalent cause of cancer death worldwide [1]. The two most common histological types of EC are esophageal adenocarcinoma (EAC) and esophageal squamous cell carcinoma (ESCC). While EAC is most prevalent in North America and Europe [2], ESCC is predominant in China, Japan, and South Korea. More than 90% of ESCC cases occur in China [3,4]. The survival rates of EC remain extremely low in most countries, ranging from 10% to 30% five years post-diagnosis [5]. In 2020 alone, there were 604,100 incidences and 544,100 deaths of EC worldwide, which corresponds to age-standardized incidence and mortality rates of 6.3 and 5.6 per 100,000, respectively. Both rates are two to three times higher in men (9.3 and 8.2) than in women (3.6 and 3.2) [6]. According to the projections by GLOBOCAN 2020, the incidences of EC in 2030 and 2040 will increase 31.4% and 33.0% from 2020, reaching approximately 739,666 and 987,723, respectively; the EC-related deaths in 2030 and 2040 will increase 63.5% and 68.0% from 2020, reaching approximately 723,466 and 914,304, respectively [1]. The incidence and mortality rates also vary substantially across countries and populations due to the prevalence of underlying risk factors and the distribution of different subtypes. In comparison to Western Africa and Central America, which recorded the lowest rates, Eastern Asia, Southern Africa, and Eastern Africa recorded the highest rates. These findings highlight the urgency of adopting effective prevention and early detection strategies to tackle the growing burden of esophageal cancer.

Surgery remains to be one of the most effective therapeutic approaches for EC, with perioperative chemotherapy or chemotherapy–radiation administered to locally advanced EC patients. However, not all the patients are eligible for resection [7,8,9,10,11]. Compared with surgery alone, neoadjuvant chemotherapy (NAC) following surgery increases the survival rate of locally advanced ESCC, owing to its comprehensive treatment strategy [12,13,14]. It has become a standard treatment modality recommended by the National Comprehensive Cancer Network (NCCN) guidelines. However, the patients’ responses to NAC are heterogeneous, and NAC fails in roughly half of the patients. For example, paclitaxel (PTX) is an effective chemotherapeutic agent for treating advanced ESCC, but drug resistance often emerges and impairs its clinical efficacy [15]. The mechanisms contributing to the development of resistance are completely unknown [16]. Therefore, the identification of underlying biomarkers associated with NAC holds significant potential for advancing our understanding of chemo-resistance mechanisms and developing novel therapeutic targets for patients with ESCC. By leveraging such biomarkers, we may be able to develop more personalized and effective treatment strategies to improve clinical outcomes for ESCC patients.

C-Terminal Binding Protein 1 (CtBP1) is a transcriptional co-repressor that represses numerous cellular processes such as apoptosis [16]. By repressing a broad array of tumor suppressors, CtBP1 promotes cancer cell proliferation, migration, invasion, and resistance to apoptosis [17]. Recent studies have shown that CtBP1 is overexpressed in multiple cancers to profoundly influence cellular phenotypic plasticity and stem cell pathways, which drives epithelial-to-mesenchymal transition and causes genome instability [16,18]. Investigating the precise roles of these corepressors in cancer development can facilitate the identification of novel drug targets, a better understanding of the complex regulatory networks that control gene expression in cancer cells, and ultimately the development of more effective treatments (clinical translation) to improve patient outcomes.

In this paper, we developed a treatment strategy that combines Clustered Regularly Interspaced Short Palindromic Repeats (CRISPR)/CRISPR-associated protein 9 (CRISPR/Cas9) genome editing with conventional chemotherapy. We first utilized CRISPR/Cas9 to knockout (KO) CtBP1 in chemoresistant ESCC cells, preventing it from producing a functional protein. This led to the increased sensitivity of the ESCC cells to the anticancer drug paclitaxel. We also examined the impact of CtBP1-KO on cell viability, chemosensitivity, and metastatic potential. This strategy may offer a promising avenue for developing new therapies to combat chemoresistance in ESCC.

## 2. Results

### 2.1. Established PTX-Resistant ESCC Cell Lines

Chemoresistance is a significant challenge in the treatment of many cancers, including ESCC. To investigate chemoresistance, we established PTX-resistant cell lines (KYSE-50/PTX and TE-1/PTX) through the gradual exposure of parental cells (KYSE-50 and TE-1) to increasing doses of paclitaxel. These PTX-resistant cells served as the foundation for generating different experimental groups, including scramble gRNA as negative control (Ctrl) Clones, the Paclitaxel alone group (PTX), the KO Clones alone (KO), and the KO Clones with Combination Paclitaxel Treatment (KO + PTX). These distinct groups were employed in three metastatic potential assays: a cell migration assay, a cell invasion assay, and a wound healing assay. The objective of these assays was to unveil the influence of CtBP1 on the metastatic potential of PTX-resistant ESCC cells.

### 2.2. CtBP1 Is Notably Expressed in Esophageal Squamous Cell Carcinoma (ESCC) Cells

**Figure 1:**
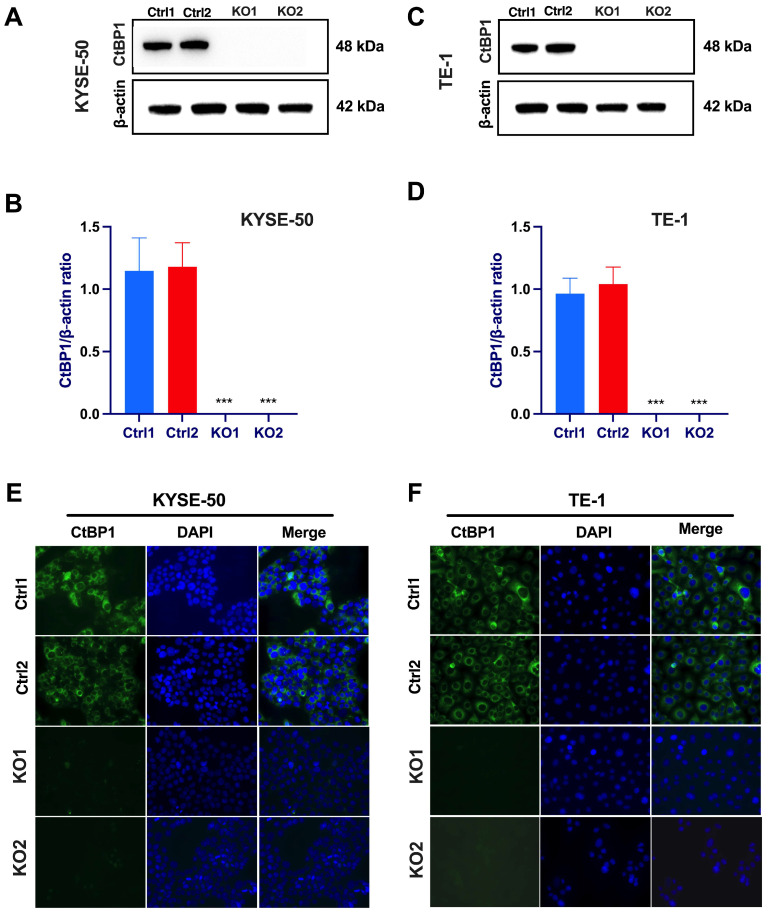
ESCC cell lines exhibit elevated CtBP1 expression. Western blot analysis was conducted to evaluate CtBP1 protein expression levels in KYSE-50 (**A**,**B**) and TE-1 (**C**,**D**) cell lines. The cell lysates originated from two distinct sets of clones, scramble gRNA as a negative control (Ctrl1 and Ctrl2 clones) and CtBP1 KO clones (KO1 and KO2 clones). The expression levels of CtBP1 were normalized using beta-actin as a loading control. A statistical analysis was performed using a one-way analysis of variance (ANOVA), followed by Tukey’s test for multiple comparisons. All experiments were performed in triplicate. Tukey’s test was utilized to assess significant differences between individual groups, with significance levels denoted as *** *p* < 0.001. A *p*-value of < 0.05 was considered indicative of significance. Immunofluorescence staining of CtBP1 in KYSE-50 cells (**E**) and TE-1 cells (**F**) were performed on CtBP1 KO clones (KO1 and KO2 clones), with scramble sgRNA as control (Ctrl1 and Ctrl2). The cytoplasmic distribution of CtBP1 was visualized using an anti-CtBP1 antibody (1:100) and Alexa Fluor™ Plus 488-conjugated secondary antibody (green) (10 μg/mL). The nuclei were counterstained with VECTASHIELD^®^ Antifade Mounting Medium containing DAPI (blue). Images in both panels were captured at 20× magnification.

We assessed the expression levels of CtBP1 in human ESCC cells, specifically, the KYSE-50 and TE-1 cell lines. Our Western blotting results (Figure 1A–D) and immunofluorescence assay (Figure 1A,F) highlight the substantial expression of CtBP1 in ESCC cells. These observations align with previous studies that have shown the involvement of CtBP1 in various cancers, including breast, lung, and prostate cancers [18,19,20,21]. This suggests that CtBP1 may play a role in the development or progression of ESCC. Our study provides the initial evidence of CtBP1 expression in human ESCC cells, indicating its potential relevance to ESCC and broader tumorigenesis.

### 2.3. CRISPR/Cas9-Mediated Suppression of Oncogenic CtBP1 Expression in Paclitaxel-Resistant ESCC Cells

To uncover the functional consequences of CtBP1 expression in ESCC, we employed a CRISPR-mediated knockout strategy. By utilizing two CtBP1-specific single guide RNAs (sgRNAs) along with two scramble sequences as controls (Ctrl), we successfully disabled CtBP1 expression in PTX-resistant cell lines. Western blot analysis (Figure 1A–D) confirmed the successful deletion of CtBP1. Further validation of our results was achieved through an immunofluorescence assay (Figure 1E,F), highlighting the absence of CtBP1 expression within the CtBP1 knockout cells. Through this dual-validation approach, utilizing two distinct sgRNAs and two scramble sequences, we attribute the observed effects specifically to CtBP1 deletion.

### 2.4. CtBP1 Knockout Promotes Paclitaxel Sensitivity and Inhibits Proliferation In Vitro

**Figure 2:**
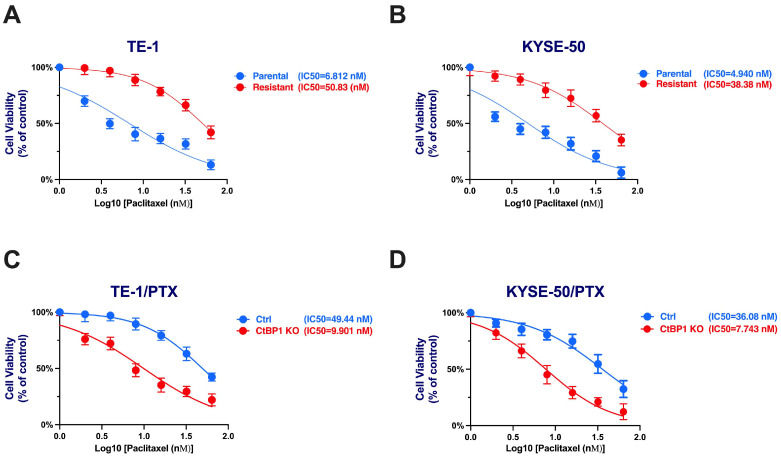
Dose–response curves for parental and PTX-resistant cell lines highlighting sensitivity disparities. (**A**) TE-1. (**B**) KYSE-50. (**C**,**D**) TE-1 and KYSE-50 categorized into Scramble control (Ctrl) and CtBP1 KO (Knockout) groups, highlighting treatment-induced changes in drug sensitivity. Experiments were conducted in triplicate, and a Student *t*-test was used to assess the difference between Ctrl and CtBP1 KO, as well as between parental and resistant groups. *p*-values < 0.05 were considered significant.

Cell viability was utilized to evaluate the cytotoxicity of paclitaxel in ESCC cells among different treatment groups. PTX-resistant cell lines demonstrated notable resistance in comparison to their parental counterparts. The IC50 (half-inhibitory concentration) values for the parental TE-1 cell line and TE-1/PTX were 6.812 and 50.83 nM (*p* = 0.0019), respectively. Similarly, the parental KYSE-50 displayed an IC50 of 4.940 nM, while the KYSE-50/PTX exhibited an IC50 of 38.38 nM (*p* = 0.0013) (Figure 2A,B). 

Furthermore, we investigated the impact of CtBP1 KO on paclitaxel sensitivity by comparing it to the control group. Our dose–response analysis illustrated a remarkable reversal of resistance in the CtBP1 KO group in comparison to the Ctrl group. The IC50 values for paclitaxel in the CtBP1 KO group (TE-1/PTX) were 9.901 nM vs. Ctrl 49.44 nM (*p* = 0.0033) and (KYSE-50/PTX) 7.743 nM vs. Ctrl 36.08 nM (*p* = 0.0086) (Figure 2A–D). The IC50 values were significantly lower in the CtBP1 KO group compared to the control group in both TE-1/PTX and KYSE-50/PTX cells. These results highlight the potent effectiveness of CtBP1 KO in an ESCC-resistant cell model. Editing CtBP1 may offer a promising avenue for targeted therapies.

**Figure 3:**
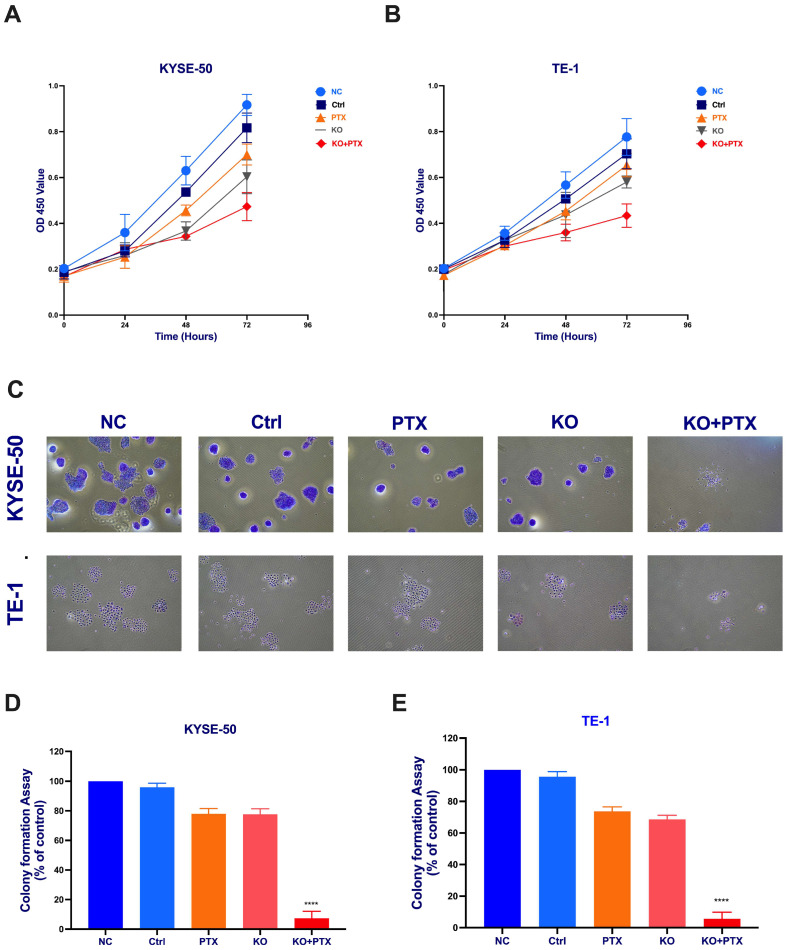
Synergistic combination of CRISPR/Cas9-mediated CtBP1 knockout and PTX treatment inhibits cell proliferation. Parental paclitaxel-sensitive KYSE-50 (**A**) and TE-1 (**B**) cells were treated with DMSO (NC), scramble sgRNA (Ctr), 10 nM PTX, CtBP1 KO, and CtBP1 KO + PTX, respectively. Cell proliferation was assessed using a CCK-8 assay at 0, 24, and 48 h post-treatment. (**C**) Representative photograph of the colonies in KYSE-50 and TE-1 cells lines showing resistance colonies in the culture dishes. Images in both panels were captured at 10× magnification. (**D**) Quantification of the colony formation assay in parental and PTX-resistant KYSE-50. (**E**) TE-1 cells after different treatments. The data are presented as mean ± standard deviation (SD) and are derived from three independent experiments. For statistical analysis, a one-way analysis of variance (ANOVA) was initially conducted, followed by Tukey’s test for multiple comparisons. Tukey’s test was applied to discern notable distinctions between individual groups, with the degrees of significance represented as **** *p* < 0.0001. All experimental procedures were meticulously conducted in triplicate. A *p*-value less than 0.05 was considered indicative of statistical significance.

We further evaluated the role of CtBP1 in cell proliferation and colony formation by conducting a series of experiments on genetically engineered CtBP1-deficient ESCC cell lines. First, we examined the effect of the CRISPR/Cas9-mediated knockout of CtBP1 on cell growth and chemosensitivity to paclitaxel. We conducted a proliferation assay using the CCK8 reagent to measure the cell population after treatment and found that only the KO + PTX group showed significant inhibition of proliferation compared to the normal control (NC), suggesting that CtBP1 plays a critical role in promoting cell growth in ESCC cells (Figure 3A,B). Furthermore, we also assessed the colony-forming ability of the different treatment groups using a colony-formation assay. Our results showed that the colony-forming ability of the KO + PTX group was inhibited by paclitaxel combined with CtBP1 KO (Figure 3C,D). These findings suggest that CtBP1 may play a key role in the chemosensitivity of ESCC cells to paclitaxel, and that targeting CtBP1 may be a promising strategy for inhibiting colony formation and the proliferation of ESCC cells.

Our results are consistent with previous studies demonstrating that CtBP1 promotes cell proliferation and inhibits apoptosis in cancer cells [18]. Furthermore, our findings support previous research showing that CtBP1 knockdown enhances chemosensitivity in other cancer types [22]. Our study provides novel insights into the molecular mechanisms of ESCC and highlights the potential of CRISPR technology as a powerful tool for investigating gene function in cancer.

### 2.5. CtBP1 Expression Drives Cell Migration and Invasion in ESCC Cells

CtBP1, a well-established gene expression regulator, has been implicated in the metastatic behavior of cancer cells. To investigate the impact of CtBP1 expression on ESCC cell metastatic behavior, we conducted a series of assays encompassing migration, invasion, and wound healing dynamics. Migration and invasion capabilities were evaluated through Boyden chamber assays, while wound healing was evaluated by the collective cell migration assays.

**Figure 4:**
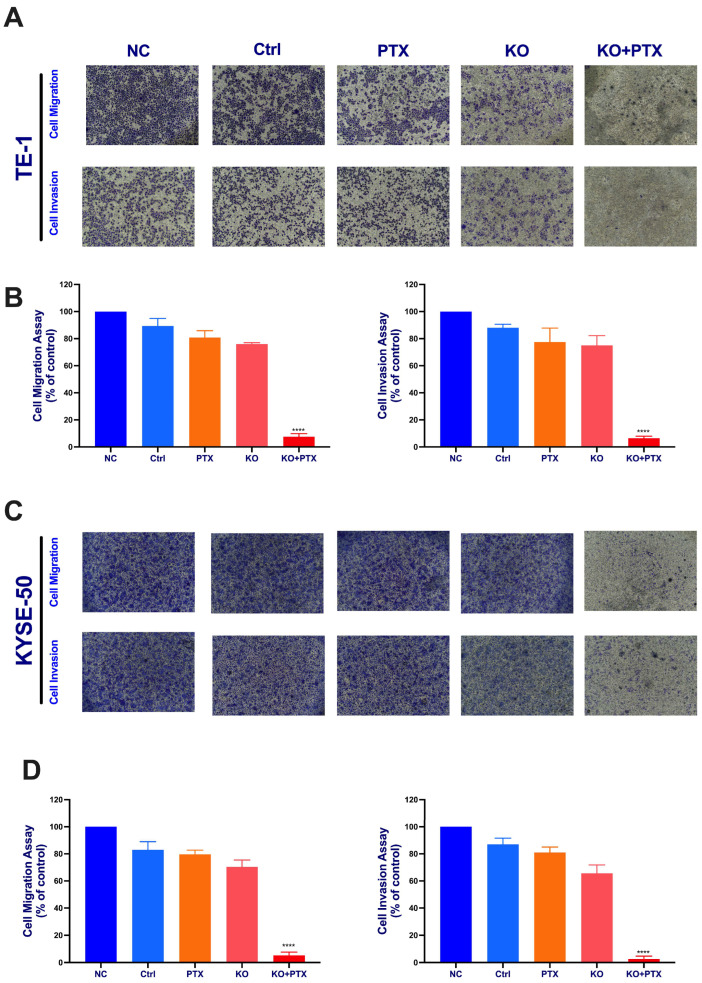
CtBP1 knockout suppresses metastatic potential of ESCC cells. (**A**) Representative images of TE-1 cells migration and invasion. (**B**) Quantification of TE-1 cells migration and invasion. (**C**) Representative images of KYSE-50 cells migration and invasion. (**D**) Quantification of KYSE-50 cells migration. The images in panels (**A**,**C**) were captured at 10× magnification. All experiments were performed in triplicate. Statistical analysis was performed using ANOVA followed by Tukey’s test for multiple comparisons. Levels of significance are denoted by the notation **** *p* < 0.0001. A *p*-value of less than 0.05 was considered significant.

**Figure 5:**
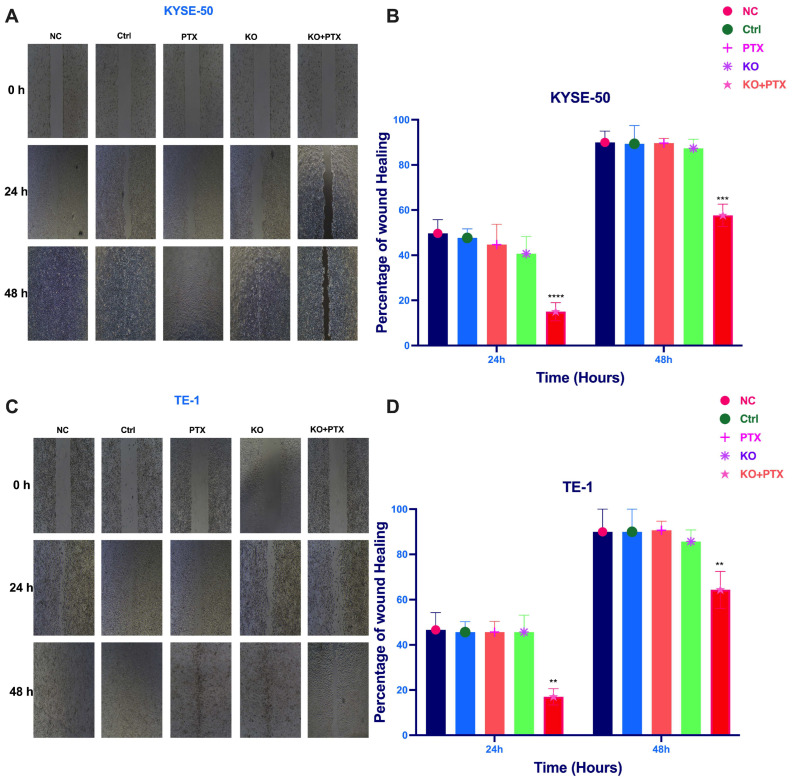
Decelerated cell migration in CtBP1 knockout ESCC cells. (**A**) Representative bright-field images of KYSE-50 cells treated with KO + PTX showing a decrease in cell invasion into the cell-free region compared to the control group. (**B**) Summary bar graph illustrating the percentage of wound closure at indicated time points during the scratch wound assay in KYSE-50 cells. Treatment with KO + PTX resulted in a significant reduction in the wound closure percentage compared to untreated cells. (**C**) Representative bright-field images of TE-1 cells treated with KO + PTX showing a decrease in cell invasion into the cell-free region compared to untreated cells. (**D**) Summary bar graph illustrating the percentage of wound closure at indicated time points during the scratch wound assay in TE-1 cells. The graph shows the percentage of wound closure at different time points after treatment compared to controls. The images in panels (**A**,**C**) were captured at 10× magnification. Data are presented as mean ± SD of three independent experiments, each performed in triplicate. Statistical significance was assessed using a two-tailed Student’s *t*-test, and significance levels were indicated by the notation ** *p* < 0.01, *** *p* < 0.001, and **** *p* < 0.0001. A *p*-value less than 0.05 was considered statistically significant.

Given that the IC50 of parental cell lines is below 10 nm, we opted for a standardized dose to treat all groups and observe the effects. All treatment groups were subjected to 10 nM paclitaxel treatment. These cells were divided into five experimental groups: normal control (NC), scramble sgRNA (Ctrl), PTX treatment alone group, KO alone, and the synergistic KO combined with paclitaxel treatment (KO + PTX). Notably, our results show that the synergistic combination of paclitaxel and CtBP1 KO reversed resistance and significantly inhibited cell migration and invasion in parental sensitive and PTX-resistant cells (Figure 4). Additionally, the wound healing assays demonstrated a deceleration in cell migration (Figure 5A–D). Specifically, only the KO + PTX group exhibited a significant reduction in cell migration and invasion, suggesting that CtBP1 plays a crucial role in promoting these processes in ESCC cells. Additionally, the KO group also exhibited a significant delay in wound closure, further supporting the role of CtBP1 in promoting cell migration and invasion.

These findings provide valuable insights into the biological behavior of ESCC cells and underscore the potential significance of targeting CtBP1 as a therapeutic strategy for this malignancy.

## 3. Discussion

Paclitaxel resistance and tumor recurrence are common in patients with locally advanced esophageal squamous cell carcinoma (ESCC) undergoing neoadjuvant chemotherapy. To improve prognosis, a better understanding of the molecular mechanisms of paclitaxel resistance in ESCC is necessary. In this study, we demonstrated that ESCC cells expressed significantly higher levels of CtBP1 and were associated with tumorigenesis and poor response to paclitaxel treatment. Using a CRISPR-directed gene editing technique, we successfully disabled CtBP1 alleles in paclitaxel-resistant ESCC cells, and observed that CtBP1 KO suppressed oncogenic expression, inhibited proliferation, migration and invasion in ESCC cells, and sensitized them to paclitaxel. Paclitaxel is a microtubule-targeting agent that inhibits cell proliferation, stabilizes cellular microtubules, and induces apoptosis [23]. Recently, paclitaxel, along with other chemotherapy drugs, has been investigated for its effectiveness in neoadjuvant therapy for ESCC, and has been shown to provide survival benefits for patients with advanced ESCC. However, it is also characterized by some key limitations due to paclitaxel resistance, which is a major component of many induction chemotherapy regimens for ESCC [15,24,25]. Therefore, the identification of the biomarkers associated with NAC can provide valuable insights into the mechanisms underlying chemoresistance in ESCC. This knowledge can then be used to develop new and more effective therapies for patients suffering from lethal malignancy. By understanding the specific biomarkers that contribute to chemoresistance, researchers can design targeted therapies that inhibit these biomarkers and overcome treatment resistance. This can lead to improved outcomes and survival rates for patients with ESCC.

In our previous research, we conducted a comprehensive investigation of biomarkers for predicting lymphatic metastatic recurrence and prognosis in ESCC [26,27]. Our study involved an RNAi-based approach to knockdown the expression of oncogenes and tumor suppressor genes in ESCC and gastric carcinoma [28,29,30]. We also investigated the application of RNAi techniques to overcome drug resistance associated with antimicrotubule drugs such as paclitaxel and vincristine in ESCC [31]. In the present study, we were able to precisely manipulate gene expression in ESCC cell models by using the CRISPR/Cas9 genome editing technique. The high precision of CRISPR/Cas9 is one of its key advantages over other methods of gene manipulation [32,33]. By precisely manipulating the target gene’s expression, we were able to investigate its potential role in drug resistance in ESCC, leading to important insights into the molecular mechanisms underlying this phenomenon. These findings could potentially contribute to the development of new treatment strategies for ESCC. The proper functioning of cells relies on the precise regulation of many transcription factors in terms of timing and location to maintain a delicate balance between cell proliferation and differentiation. Any disruption to the regulation of these transcription factors, especially those that can influence entire gene networks, may contribute to the development of tumorigenic behavior [34]. Transcriptional corepressors are frequently subjected to changes due to loss- or gain-of-function mutations in cancer, which can lead to an imbalance in transcriptional regulation. Consequently, corepressor proteins have emerged as an essential component of cancer etiology [35,36]. 

Based on our previous works, the present study focused on a novel gene CtBP1, which is associated with “cancer hallmarks” and has been observed to promote tumorigenesis when overexpressed [18]. It is also known that CtBP1 plays a critical role in regulating cell proliferation and apoptosis in a variety of cancers, including breast, ovarian, and osteosarcoma [17,37,38]. Our research found the strong expression of CtBP1in ESCC cells, and functional studies revealed that the altered expression of this gene contributes to the metastatic potential of ESCC. Previous studies showed that as a transcriptional regulator, CtBP1 also plays a crucial role in developmental processes and can enhance malignant growth in adult tissues. CtBP1 has been shown to promote epithelial–mesenchymal transition, a process that plays a critical role in cancer metastasis, by repressing the expression of E-cadherin, which is a vital mediator of cell–cell adhesion [39]. CtBP1 is overexpressed in various types of cancer such as breast, lung, liver, and colorectal cancers, and its upregulation is linked to worse clinical outcomes, including increased tumor growth, invasion, and metastasis, as well as poor response to chemotherapy and radiation therapy [18,40]. Previous research has demonstrated that targeting the CtBP1–FOXM1 complex with small molecules represents a promising approach to overcoming MDR1-mediated chemoresistance in osteosarcoma cancer stem cells [36]. It is noteworthy to consider the potential relationship between CtBP1 and microtubule dynamics, although empirical evidence linking the two remains limited.

To investigate the mechanisms of drug resistance in cancer cells, it is crucial to develop drug-resistant cancer cell lines. This is typically achieved by gradually exposing cancer cells to increasing concentrations of specific drugs over an extended period [41]. Previously, paclitaxel-resistant cell lines have been established for this purpose, and several studies have focused on understanding the underlying mechanisms of resistance [42,43,44,45].

To comprehensively investigate the role of the CtBP1 gene in chemoresistance in ESCC, we generated two PTX-resistant cell lines (KYSE-50/PTX and TE-1/PTX) by gradually exposing them to escalating concentrations of paclitaxel. The resultant cell lines exhibited significant resistance to paclitaxel treatment, providing a valuable model for studying chemoresistance in ESCC. To deepen our understanding of the role of CtBP1 in paclitaxel resistance in ESCC, we employed an advanced and highly precise technique known as CRISPR-Cas9 genome editing. Using this technique, we were able to specifically target and KO the CtBP1 gene in ESCC cells, creating paclitaxel-resistant cell models that lacked the expression of CtBP1. This allowed us to study the effects of CtBP1 KO on the molecular pathways involved in paclitaxel resistance, as well as to investigate potential therapeutic targets that may be impacted by the loss of CtBP1 expression. By employing cutting-edge technologies such as CRISPR-Cas9 genome editing, we aim to make significant strides towards understanding the mechanisms of cancer drug resistance and developing effective treatments for patients with ESCC. Upon disabling the CtBP1 gene in these resistant cell lines, we observed enhanced sensitivity to paclitaxel in the CtBP1 KO group (Figure 2). We also found a significant reduction in cell viability (Figure 3A, B), indicating that the CtBP1 gene plays a crucial role in conferring chemoresistance to these cell lines. Additionally, we found that the KO of the CtBP1 gene also inhibited the colony formation of resistant cell lines, providing further evidence of the significant reversal of chemoresistance (Figure 3C–E). To determine the metastatic potential, we conducted experiments using cell migration/invasion and wound healing assays. Our results show the inhibition of cell migration and invasion in CTBP1-deficient cell lines treated with paclitaxel (Figure 4 and Figure 5), indicating that the CtBP1 gene may also play a role in regulating the metastatic potential of ESCC cells. These findings are consistent with previous studies that have identified CtBP1’s significant role in development and oncogenesis [20].

Our findings suggest that CtBP1 expression plays a crucial role in the development of chemoresistance and tumorigenesis in ESCC. Targeting CtBP1 expression using CRISPR-Cas9-mediated gene editing holds great promise as a novel therapeutic strategy for inhibiting the growth and progression of ESCC tumors and sensitizing cells to paclitaxel. Additionally, the limited expression of CtBP1 in most adult tissues and its ability to reactivate developmental programs when markedly expressed make it an attractive target for cancer therapy. Moreover, further research directed at expanding our current understanding of transcriptional corepressors in oncogenesis could significantly impact the development of novel therapeutic approaches. While our study primarily focuses on investigating CtBP1’s role in acquired paclitaxel resistance, we recognize the intriguing potential for mechanistic connections with microtubule dynamics. It is conceivable that CtBP1’s functions might intersect with these processes. For instance, CtBP1 has been implicated in epithelial–mesenchymal transition (EMT), a phenomenon associated with cytoskeletal rearrangements and changes in cellular motility [39]. Furthermore, CtBP1’s differential regulation of genomic stability and the DNA repair pathway could indirectly impact microtubule stability and function [46]. Microtubules constitute fundamental components of the cell’s cytoskeleton, crucial for processes such as cell division, intracellular transportation, and the maintenance of cell shape [47]. Notably, paclitaxel resistance often arises due to alterations in microtubule dynamics [48]. While these connections remain speculative, they offer compelling avenues for future exploration.

Our study provides valuable insights into the potential of targeting CtBP1 expression as a promising therapeutic approach for ESCC. Furthermore, highlighting the importance of investigating potential links between CtBP1 and microtubule dynamics offers avenues for exploring novel resistance mechanisms and gaining a deeper understanding of CtBP1’s implications in cancer biology.

## 4. Materials and Methods

### 4.1. Chemicals and Reagents

The chemicals and reagents used in this study were obtained from various commercial suppliers. Paclitaxel (PTX) was procured from Med Chem Express (Shanghai, China), while Giemsa dye was obtained from Biosharp Biotechnology Co., Ltd. (Hefei, China). Polybrene^®^ and dimethyl sulfoxide (DMSO) were purchased from Sigma-Aldrich (St Louis, MO, USA). The VEC-TASHIELD^®^ Antifade Mounting Medium with DAPI was obtained from Vector Laboratories (#H-1200, Burlingame, CA, USA). The Transwell™ chambers and Corning^®^ BioCoat™ Matrigel^®^ Invasion Chamber were both acquired from Corning (Corning, NY, USA).

### 4.2. Cell Lines and Cell Culture

The human ESCC cell lines TE-1 and KYSE-50 were obtained from the National Collection of Authenticated Cell Cultures and were cultured in an incubator at 37 °C and 5% CO_2_. Paclitaxel-resistant cell lines KYSE-50 and TE-1 were generated from the parental cell lines by gradually increasing the concentration of paclitaxel (Med Chem Express, CHINA) in accordance with established protocols [41]. Paclitaxel-resistant cell lines (KYSE-50/PTX and TE-1/PTX) were maintained in culture with the addition of paclitaxel (10 nM) to sustain the resistant phenotype [49]. The cells were routinely cultured and maintained in Roswell Park Memorial Institute (RPMI) 1640 Medium supplemented with 10% (*v*/*v*) fetal bovine serum (FBS), 50 I.U./mL penicillin, and 50 μg/mL streptomycin (Gibco, Invitrogen Co., Carlsbad, CA, USA) and 2.5 μg/mL PlasmocinTM (Invivogen, San Diego, CA, USA). The cells were treated with 0.25% trypsin (Gibco, Invitrogen Co., Carlsbad, CA, USA) and subculture at a 1:8 split ratio and were then cryopreserved in Corning^®^ Cryogenic Vials (Corning Inc, Corning, NY, USA) containing 10% (*v/v*) dimethyl sulfoxide (DMSO) (Sigma-Aldrich, St Louis, MO, USA), 70% (*v/v*) FBS, and 10% (*v/v*) DMEM. For short-term storage up to 30 days, the cells were stored in a low-temperature freezer at −80 °C. For long-term storage, the cells were placed in liquid nitrogen (LN2).

### 4.3. Antibodies

The antibodies used in this study are as follows: Anti-CtBP1 (1:5000, Abcam, Cambridge, MA, USA); β-Actin Rabbit mAb (High Dilution) (1:100,000, Abclonal Technology Co., Ltd., Wuhan, China); Goat Anti-Rabbit IgG H, and L (HRP) (1:500, Abcam, Cambridge, MA, USA); and Alexa Fluor™ Plus 488 conjugated secondary antibodies (Gibco, Invitrogen Co., Carlsbad, CA, USA).

### 4.4. Guide RNA Design and Construction

Single guide RNAs (sgRNAs) targeting CtBP1 were designed following https://www.benchling.com/crispr (accessed on 15 January 2021), with the guide sequences selected based on the on-target score. The on-target and off-target scores were calculated using algorithms developed by Doench et al. [50,51]. sgRNAs (Table 1) with higher on-target and off-target scores were selected and validated using online sgRNA design verification tools.

After design validation, sgRNA target sequences were synthesized by Genewiz Inc. (Suzhou, China) and cloned into the LentiCRISPR v2 backbone with BsmBI, following the standard protocol described in [52]. Both sgRNAs efficiently knocked out CtBP1 gene expression. We selected the best one (AATCACTGAAGCCTGCGTCG) for the downstream experiments. For negative control, two scrambled sgRNA sequences (Table 1) were used. CtBP1 sgRNA KO clone and Scramble sgRNA (Crtl) were used for subsequent experiments.

LentiCRISPRv2 was a gift from Feng Zhang (Addgene plasmid #52961; http://n2t.net/addgene:52961 (accessed on 15 January 2021); RRID: Addgene_52961) (Figure S1). Lentivirus was generated in HEK 293T cells using Polyethylenamine (#408727, Sigma-Aldrich), 2nd generation lentiviral packaging plasmid (psPAX2) (Figure S2), and pVSV-G envelope-expressing plasmid (pMD2.G) (Figure S3). pMD2.G (Addgene plasmid #12259; http://n2t.net/addgene:12259 (accessed on 15 January 2021); RRID: Addgene_12259) and psPAX2 (Addgene plasmid #12260; http://n2t.net/addgene:12260 (accessed on 15 January 2021); RRID: Addgene_12260) were gifts from Didier Trono.

The lentivirus generated with LentiCRISPRv2 was used to transduce ESCC cells with polybrene transfection reagent (MerckMillipore, #TR-1003-G), and selection was performed with puromycin (1 μg/mL) for two weeks prior to immunofluorescence staining (IF) and Western blotting (WB) experiments. We validated the effectiveness of two scrambled sgRNAs and two CtBP1-targeting sgRNAs through Western blotting and immunofluorescence assays. For subsequent experiments, we chose the most effective control clone (Scramble sgRNA, Ctrl) and knockout clone (CtBP1 sgRNA KO) to conduct further downstream analyses.

### 4.5. Creation of CtBP1-KO Clonal ESCC Cell Lines Using a CRISPR-Directed Gene-Editing Approach

We utilized a CRISPR-directed gene editing approach to functionally disable CtBP1 alleles in ESCC cells. sgRNAs-targeting CtBP1s were designed using the web-based platform called Benchling (https://www.benchling.com/crispr (accessed on 15 January 2021)). We selected guide sequences based on their on-target and off-target scores, which were calculated by Benchling using algorithms developed by Doench et al. and Hsu et al. [50,51]. Both scores ranged from 0 to 100, with higher scores being better. The on-target score of an sgRNA design does not directly represent the cleavage efficiency of Cas9. Instead, it is a predictive score of the likelihood that the sgRNA will bind specifically to the intended target sequence in the genome [53]. On-target scores are calculated based on a variety of factors, including the proximity of the target sequence to the PAM site, the GC content and sequence complexity of the target region, and the potential for off-target effects in non-coding regions of the genome. The off-target score of an sgRNA design represents the inverse probability of Cas9 off-target. A higher score means the sequence is less likely to bind to sequences in the rest of the genome.

To validate the precision and efficacy of our design, we employed the IDT CRISPR-Cas9 guide RNA design checker (https://sg.idtdna.com/site/order/designtool/index/CRISPR_SEQUENCE) and the SYNTHEGO guide design validation tools (https://design.synthego.com/#/validate). Examples of potential off-target sequences can be found in Figure S4. The specific sequences for both the sgRNA target and the scramble control are provided in Table 1. The chosen sgRNAs were synthesized by Genewiz Inc. (Suzhou, China) and then integrated into the lentiCRISPR v2 vector (illustrated in Figure S1) using BsmBI enzyme. The linear map of the CRISPR/Cas9 vector, showing the sgRNA’s position, is depicted in Figure S5A. Notably, the guide was designed to target the primary transcript of the CtBP1 gene (ENST00000382952) in Homo sapiens, as visualized in Figure S5B.

The lentiviral titer was produced using the second-generation lentiviral packaging plasmid (psPAX2) (Figure S2) and the pVSV-G envelope-expressing plasmid (pMD2.G) (Figure S3). ESCC cells were transfected with polybrene reagent, and selection was performed using puromycin (1 μg/mL).

### 4.6. Immunofluorescence

Cells were cultured in an 8-well Lab-tek II chamber slide (Nalge Nunc International, Rochester, NY, USA) for 2–3 days, washed with 1X phosphate-buffered saline (1XPBS), and fixed with 4% paraformaldehyde for 15 min at room temperature. After fixation, the cells were rinsed with 1X PBS three times for 5 min each time at room temperature and permeabilized for 15 min in permeabilization buffer (1X PBS/0.2% Triton X-100). Next, the cells were incubated in blocking buffer (1X PBS/5% normal serum/0.3% Triton X-100) for 1 h. After blocking, the cells were incubated overnight at 4 °C with anti-CtBP1 antibody (1:100, Abcam, Cambridge, MA, USA) diluted in blocking buffer. Following three washes in 1X PBS for 5 min each time, the specimens were incubated for 1 h at room temperature with Alexa Fluor™ Plus 488 conjugated secondary antibody (10 μL/mL) (Invitrogen Co., USA) diluted in antibody dilution buffer (1X PBS/1% bovine serum albumin/0.3% Triton X-100). After incubation with the secondary antibody, the chamber slides containing cells were washed three times with 1X PBS and mounted in Vectashield antifade mounting media (H-1200, Vector Laboratories, Burlingame, CA, USA), and observed with a fluorescence microscope.

### 4.7. Western Blotting

Western blotting was performed as described previously [54]. ESCC cells were cultured in triplicate in Costar^®^6-well Clear TC-treated Multiple Well Plates (Corning Inc., Corning, NY, USA) and treated with either DMSO or drugs of varying concentrations for 24 h at 37 °C and 5% CO_2_. Upon reaching confluence, cells were washed with Gibco^TM^ phosphate-buffered saline (PBS) antibodies (Gibco, Invitrogen Co., Carlsbad, CA, USA) and homogenized with 400 μL of M-PER^®^ Reagent (Mammalian Protein Extraction Reagent, Thermo Fisher Scientific, Waltham, MA, USA) supplemented with 10 μL/mL HaltTM Protease and Phosphatase Inhibitor Cocktail (Thermo Fisher Scientific, Waltham, MA, USA). The samples were incubated on ice, spun at ~14,000× *g* for 10 min, and the clear supernatant was collected. Protein concentrations were quantified using a Pierce^TM^ BCA Protein Assay (Thermo Fisher Scientific, Waltham, MA, USA). For immunoblotting, 20 micrograms of each protein sample were denatured at 100 °C for 10 min and loaded into a gel for separation by high-performance precast mini poly-acrylamide gels 4–20% (ExpressPlus PAGE Gel) (Genscript Inc., Piscataway, NJ, USA). Protein transfer was carried out using Immo-bilon^®^-P PVDF membrane (polyvinylidene fluoride Membrane, Pore size, 0.45 μm) (EMD Millipore Co., Darmstadt, Germany). The membranes were blocked in Can Get Signal^®^ Immunoreaction Enhancer Solution (TOYOBO Co. Ltd., Osaka, Japan) for two h at room temperature and then incubated with primary antibody overnight at 4 °C. All primary antibodies were diluted in Can Get Signal^®^ Solution 1 (TOYOBO Co. Ltd., Osaka, Japan). The membranes were washed with Tris-Buffered Saline with 0.1% Tween 20 (TBS-T) (Takara Bio USA, Mountain View, CA, USA) three times for 10 min. Secondary antibodies were diluted in Can Get Signal^®^ Solution 2 (TOYOBO Co. Ltd., Osaka, Japan) and incubated for 1 h at room temperature. The immunoblots were washed three times with 1XTBS-T for 30 min before being developed with ECL substrate (Clarity™ Western ECL Substrate, Bio-Rad, Hercules, CA, USA).

To quantify the band intensities of CtBP1 and β-Actin, we employed ImageJ software (Version, 1.53t; National Institutes of Health, Bethesda, MD, USA). β-Actin was used as a protein-loading control to normalize the protein concentration variation across different samples.

### 4.8. Cell Proliferation Assay

The study aimed to evaluate cell proliferation in both parental and PTX-resistant cell lines, specifically TE-1/PTX and KYSE-50/PTX. To comprehensively analyze the effects of CtBP1 knockout and paclitaxel treatment on cell proliferation, distinct groups were established: normal control (NC), scramble gRNA as a negative control (Ctrl), the paclitaxel alone group (PTX), the KO clones alone (KO), and the KO clones with combination paclitaxel treatment (KO + PTX). The NC group originated from the parental ESCC cell line, while all treatment groups were derived from paclitaxel-resistant cell lines (TE-1/PTX, KYSE-50/PTX). The normal control group served as a baseline for cell proliferation and was treated with DMSO. The PTX alone group and the KO alone group were treated with 10 nM paclitaxel to assess its impact on cell proliferation, and the PTX + KO group was additionally examined to explore the potential of this approach in overcoming paclitaxel resistance. Cells were plated at a density of 2000 cells per well in 100 µL of RPMI 1640 medium supplemented with 10% FBS in Corning^®^ 96-well Clear Flat Bottom Polystyrene TC-treated Microplates (Corning Inc., Corning, NY, USA) and incubated overnight at 37 °C with 5% CO_2_. The plates were then tested at 0, 24, and 48 h. For the CCK-8 assay, 10 μL of CCK-8 reagent was added to 100 μL of fresh medium per well, and the cells were incubated for 1 h at 37 °C. The absorbance of the wells was measured at 450 nm using a microplate reader. The viability of cells in each well was calculated as a percentage of absorbance of the experimental group compared to that of the control group. The experiment was performed in triplicate, and the results are presented as mean ± standard deviation (SD).

### 4.9. Cell Viability Assay

Cell viability was assessed using the CCK-8 (Cell Counting Kit-8™, Dojindo Molecular Technologies Inc., Kumamoto, Japan) in both parental-sensitive and paclitaxel-resistant cell variants, as well as the scramble control (Ctrl) and CtBP1 KO (Knockout) groups. Cells were cultured in 96-well plates and exposed to a range of paclitaxel concentrations (0, 2, 4, 6, 8, 16, 32, and 64 nM) for 72 h. Following incubation, 10 μL of CCK-8 reagent was added to each well and incubated at 37 °C with 5% CO_2_ for 1 h. The absorbance at 450 nm was measured using a PerkinElmer Multimode Plate Reader EnSpire. 

### 4.10. IC50 Calculation

Dose–response curves were generated using GraphPad Prism software (Prism 9 for macOS). Concentrations were expressed in a logarithmic scale to accurately model the dose–response relationship. Non-linear regression analysis was performed to fit the curves. IC50 values, representing the concentration of paclitaxel required to inhibit cell viability by 50%, were derived from the curves for all experimental groups.

### 4.11. Colony Formation Assay

Briefly, 1000 cells from each group were plated in 6-well plates (Corning Inc., Corning, NY, USA) and incubated overnight at 37 °C with 5% CO_2_. On the subsequent day, all treatment groups were exposed to 10 nM paclitaxel and cultured for a duration of 10 days, with medium replacements conducted every 3–4 days. In contrast, the control group received the same culture conditions without paclitaxel treatment. Following the culture period, the cells were fixed using 4% paraformaldehyde and subsequently subjected to Giemsa staining. The quantification of colony formation in each well was performed using a microscope.

The outcomes were depicted as mean ± standard deviation (SD). Statistical analysis encompassed the utilization of one-way analysis of variance (ANOVA), followed by Tukey’s post-hoc test for pairwise comparisons. A *p*-value below 0.05 was regarded as indicative of statistical significance.

### 4.12. Wound Healing Assay

Both the normal control group and the various experimental groups were seeded into 6-well plates, permitting the formation of confluent monolayers. Subsequently, a sterile 200 μL pipette tip was employed to create a central scratch/wound within each well. Gentle rinsing with PBS was then conducted to eliminate any dislodged cells. The addition of the appropriate medium (RPMI 1640 supplemented with 10% FBS) followed, and the cells were subsequently incubated at 37 °C with 5% CO_2_ for 24 and 48 h.

To assess the effects of treatment on cell migration, images of the scratch area are captured at t = 0 h (initial scratch area), t = 24 h, and t = 48 h. To quantify the area of the scratch, the Montpellier Ressources Imagerie (MRI) wound healing tool in ImageJ analysis software [55] was used. The migration of cells toward the scratch area was expressed as the percentage of scratch cell migration, which was calculated using the formula: Scratch Cell Migration (%) = [(A_t=0h_ − A_t=∆h_)/A_t=0h_] × 100 [56], where A_t=0h_ is the initial area of the scratch (wound) at 0 h and A_t=∆h_ is the remaining area of the scratch at a specified time point (e.g., 24 h).

### 4.13. Cell Migration Assa

Cell migration assays were conducted using Transwell™ chambers with a polycarbonate filter (6.5 mm diameter and 8.0 μm pore size, Corning, NY, USA). The upper chamber was seeded with parental controls (NC) and PTX-resistant cells, comprising all other experimental groups. Specifically, 200 μL of serum-free medium was added to the upper chamber, while the lower chamber received 750 μL of RPMI 1640 medium supplemented with 10% FBS, serving as the chemoattractant. Following a 48-h period, the medium was withdrawn, and the chambers underwent two PBS-1X washes. Migrated cells were then fixed with 4% paraformaldehyde at room temperature for 15 min, followed by three washes with PBS-1X. The cells were then permeabilized with methanol for 15 min, washed twice with PBS-1X, stained with Giemsa (Solarbio, Beijing, China) for 25 min, and washed twice with PBS-1X. To count invading cells, ten randomly selected fields of view were captured for each chamber using a digital camera attached to an inverted microscope (magnification, ×4). The experiments were performed in triplicate.

### 4.14. Cell Invasion Assay

The invasion assay was performed using Corning^®^ BioCoat™ Matrigel^®^ Invasion Chambers (Corning, NY, USA) with 8.0 µm polyester (PET) membranes. NC and PTX-resistant cells were seeded into the upper chambers with 200 μL of serum-free media. The lower chambers were filled with 750 μL RPMI 1640 medium supplemented with 10% FBS, which served as a chemoattractant. After 48 h of incubation at 37 °C with the 5% CO_2_, the medium was removed, and the chambers were washed twice with PBS-1X. Non-invading cells were removed from the upper surface of the membrane by scrubbing with a cotton-tipped swab, while invading cells were fixed with 4% paraformaldehyde for 15 min at room temperature. After washing three times with PBS-1X, the cells were permeabilized with methanol for 15 min, washed twice with PBS-1X, and stained with Giemsa (Solarbio, Beijing, China) for 25 min. The chambers were washed twice with PBS-1X again, and randomly selected fields of view (ten per chamber) were imaged using a digital camera mounted on an inverted microscope (magnification, ×4). Invading cells were then counted in each field. The experiments were performed in triplicate.

### 4.15. Statistical Analysis

Statistical analysis was performed using GraphPad Prism 9 (GraphPad Software, La Jolla, CA, USA). The data are presented as mean SD. The two-tailed unpaired Student’s test was used for the comparison of n = 2 groups. *p* < 0.05 was considered statistically significant.

## Figures and Tables

**Table 1:** SgRNA target sequences.

Name	Sequence (5′–3′)
**1-sgRNA^Scramble^**	GCACTACCAGAGCTAACTCA
**2-sgRNA^Scramble^**	GTCCACCCTTATCTAGGCTA
**1-sgRNACtBP1**	AATCACTGAAGCCTGCGTCG
**2-sgRNACtBP1**	AGTTCTGGTCGGACCTTCGA

## Data Availability

The data are contained within this article.

## Supplementary Materials

The following supporting information can be downloaded at: https://www.mdpi.com/article/10.3390/ijms241814030/s1.

## References

[1] Sung H., Ferlay J., Siegel R.L., Laversanne M., Soerjomataram I., Jemal A., Bray F. (2021). Global Cancer Statistics 2020: GLOBOCAN Estimates of Incidence and Mortality Worldwide for 36 Cancers in 185 Countries. CA Cancer J. Clin..

[2] Enzinger P.C., Mayer R.J. (2003). Esophageal cancer. N. Engl. J. Med..

[3] Zhao J., He Y.T., Zheng R.S., Zhang S.W., Chen W.Q. (2012). Analysis of esophageal cancer time trends in China, 1989–2008. Asian Pac. J. Cancer Prev..

[4] Zhou M., Wang H., Zeng X., Yin P., Zhu J., Chen W., Li X., Wang L., Wang L., Liu Y. (2019). Mortality, morbidity, and risk factors in China and its provinces, 1990–2017: A systematic analysis for the Global Burden of Disease Study 2017. Lancet.

[5] Allemani C., Matsuda T., Di Carlo V., Harewood R., Matz M., Niksic M., Bonaventure A., Valkov M., Johnson C.J., Esteve J. (2018). Global surveillance of trends in cancer survival 2000-14 (CONCORD-3): Analysis of individual records for 37,513,025 patients diagnosed with one of 18 cancers from 322 population-based registries in 71 countries. Lancet.

[6] Morgan E., Soerjomataram I., Rumgay H., Coleman H.G., Thrift A.P., Vignat J., Laversanne M., Ferlay J., Arnold M. (2022). The Global Landscape of Esophageal Squamous Cell Carcinoma and Esophageal Adenocarcinoma Incidence and Mortality in 2020 and Projections to 2040: New Estimates from GLOBOCAN 2020. Gastroenterology.

[7] Mariette C., Piessen G., Balon J.M., Van Seuningen I., Triboulet J.P. (2004). Surgery alone in the curative treatment of localised oesophageal carcinoma. Eur. J. Surg. Oncol..

[8] Roca E., Pennella E., Sardi M., Carraro S., Barugel M., Milano C., Fiorini A., Giglio R., Gonzalez G., Kneitschel R. (1996). Combined intensive chemoradiotherapy for organ preservation in patients with resectable and non-resectable oesophageal cancer. Eur. J. Cancer.

[9] Ilson D.H., van Hillegersberg R. (2018). Management of Patients with Adenocarcinoma or Squamous Cancer of the Esophagus. Gastroenterology.

[10] Backemar L., Lagergren P., Djarv T., Johar A., Wikman A., Lagergren J. (2015). Comorbidities and Risk of Complications After Surgery for Esophageal Cancer: A Nationwide Cohort Study in Sweden. World J. Surg..

[11] Yip C., Landau D., Kozarski R., Ganeshan B., Thomas R., Michaelidou A., Goh V. (2014). Primary esophageal cancer: Heterogeneity as potential prognostic biomarker in patients treated with definitive chemotherapy and radiation therapy. Radiology.

[12] Mayanagi S., Irino T., Kawakubo H., Kitagawa Y. (2019). Neoadjuvant treatment strategy for locally advanced thoracic esophageal cancer. Ann. Gastroenterol. Surg..

[13] Yang H., Liu H., Chen Y., Zhu C., Fang W., Yu Z., Mao W., Xiang J., Han Y., Chen Z. (2018). Neoadjuvant Chemoradiotherapy Followed by Surgery Versus Surgery Alone for Locally Advanced Squamous Cell Carcinoma of the Esophagus (NEOCRTEC5010): A Phase III Multicenter, Randomized, Open-Label Clinical Trial. J. Clin. Oncol..

[14] Yang H., Liu H., Chen Y., Zhu C., Fang W., Yu Z., Mao W., Xiang J., Han Y., Chen Z. (2021). Long-term Efficacy of Neoadjuvant Chemoradiotherapy Plus Surgery for the Treatment of Locally Advanced Esophageal Squamous Cell Carcinoma: The NEOCRTEC5010 Randomized Clinical Trial. JAMA Surg..

[15] Shi X., Dou Y., Zhou K., Huo J., Yang T., Qin T., Liu W., Wang S., Yang D., Chang L. (2017). Targeting the Bcl-2 family and P-glycoprotein reverses paclitaxel resistance in human esophageal carcinoma cell line. Biomed. Pharmacother..

[16] Bellesis A.G., Jecrois A.M., Hayes J.A., Schiffer C.A., Royer W.E. (2018). Assembly of human C-terminal binding protein (CtBP) into tetramers. J. Biol. Chem..

[17] Chen X., Zhang Q., Dang X., Song T., Wang Y., Yu Z., Zhang S., Fan J., Cong F., Zhang W. (2021). Targeting the CtBP1-FOXM1 transcriptional complex with small molecules to overcome MDR1-mediated chemoresistance in osteosarcoma cancer stem cells. J. Cancer.

[18] Blevins M.A., Huang M., Zhao R. (2017). The Role of CtBP1 in Oncogenic Processes and Its Potential as a Therapeutic Target. Mol. Cancer Ther..

[19] Wang R., Asangani I.A., Chakravarthi B.V., Ateeq B., Lonigro R.J., Cao Q., Mani R.S., Camacho D.F., McGregor N., Schumann T.E. (2012). Role of transcriptional corepressor CtBP1 in prostate cancer progression. Neoplasia.

[20] Chinnadurai G. (2002). CtBP, an unconventional transcriptional corepressor in development and oncogenesis. Mol. Cell.

[21] Di L.J., Byun J.S., Wong M.M., Wakano C., Taylor T., Bilke S., Baek S., Hunter K., Yang H., Lee M. (2013). Genome-wide profiles of CtBP link metabolism with genome stability and epithelial reprogramming in breast cancer. Nat. Commun..

[22] Birts C.N., Harding R., Soosaipillai G., Halder T., Azim-Araghi A., Darley M., Cutress R.I., Bateman A.C., Blaydes J.P. (2010). Expression of CtBP family protein isoforms in breast cancer and their role in chemoresistance. Biol. Cell.

[23] Jordan M.A., Wilson L. (2004). Microtubules as a target for anticancer drugs. Nat. Rev. Cancer.

[24] Yang B., Guo X., Le C., Su W., Li X., Zhang Y., Yang G., Liang W., Zheng Z., Wu J. (2022). Efficacy and Safety of Apatinib plus Neoadjuvant Chemotherapy for Locally Advanced Esophageal Squamous Cancer: A Phase II Trial. BioMed Res. Int..

[25] Liu Y., Ren Z., Yuan L., Xu S., Yao Z., Qiao L., Li K. (2016). Paclitaxel plus cisplatin vs. 5-fluorouracil plus cisplatin as first-line treatment for patients with advanced squamous cell esophageal cancer. Am. J. Cancer Res..

[26] Akhtar J., Wang Z., Yu C., Zhang Z.P., Bi M.M. (2014). STMN-1 gene: A predictor of survival in stage iia esophageal squamous cell carcinoma after Ivor-Lewis esophagectomy?. Ann. Surg. Oncol..

[27] Akhtar J., Wang Z., Jiang W.P., Bi M.M., Zhang Z.P. (2014). Stathmin overexpression identifies high risk for lymphatic metastatic recurrence in pN0 esophageal squamous cell carcinoma patients. J. Gastroenterol. Hepatol..

[28] Akhtar J., Wang Z., Zhang Z.P., Bi M.M. (2013). Lentiviral-mediated RNA interference targeting stathmin1 gene in human gastric cancer cells inhibits proliferation in vitro and tumor growth in vivo. J. Transl. Med..

[29] Akhtar J., Wang Z., Yu C., Zhang Z.P. (2014). Effectiveness of local injection of lentivirus-delivered stathmin1 and stathmin1 shRNA in human gastric cancer xenograft mouse. J. Gastroenterol. Hepatol..

[30] Habib R., Akhtar J., Taqi M., Yu C., Zhang C. (2015). Lentiviral vector-mediated survivin shRNA delivery in gastric cancer cell lines significantly inhibits cell proliferation and tumor growth. Oncol. Rep..

[31] Wang S., Akhtar J., Wang Z. (2015). Anti-STMN1 therapy improves sensitivity to antimicrotubule drugs in esophageal squamous cell carcinoma. Tumour Biol..

[32] Doudna J.A., Charpentier E. (2014). Genome editing. The new frontier of genome engineering with CRISPR-Cas9. Science.

[33] Wang H., La Russa M., Qi L.S. (2016). CRISPR/Cas9 in Genome Editing and Beyond. Annu. Rev. Biochem..

[34] Karamouzis M.V., Gorgoulis V.G., Papavassiliou A.G. (2002). Transcription factors and neoplasia: Vistas in novel drug design. Clin. Cancer Res..

[35] Vaiopoulos A.G., Kostakis I.D., Athanasoula K., Papavassiliou A.G. (2012). Targeting transcription factor corepressors in tumor cells. Cell Mol. Life Sci..

[36] Battaglia S., Maguire O., Campbell M.J. (2010). Transcription factor co-repressors in cancer biology: Roles and targeting. Int. J. Cancer.

[37] Han Y., Bi Y., Bi H., Diao C., Zhang G., Cheng K., Yang Z. (2016). miR-137 suppresses the invasion and procedure of EMT of human breast cancer cell line MCF-7 through targeting CtBP1. Hum. Cell.

[38] Chinnadurai G. (2009). The transcriptional corepressor CtBP: A foe of multiple tumor suppressors. Cancer Res..

[39] Zhang X.L., Huang C.X., Zhang J., Inoue A., Zeng S.E., Xiao S.J. (2013). CtBP1 is involved in epithelial-mesenchymal transition and is a potential therapeutic target for hepatocellular carcinoma. Oncol. Rep..

[40] Jin W., Scotto K.W., Hait W.N., Yang J.M. (2007). Involvement of CtBP1 in the transcriptional activation of the MDR1 gene in human multidrug resistant cancer cells. Biochem. Pharmacol..

[41] Wang C., Guo L.B., Ma J.Y., Li Y.M., Liu H.M. (2013). Establishment, and characterization of a paclitaxel-resistant human esophageal carcinoma cell line. Int. J. Oncol..

[42] Chen S.Y., Hu S.S., Dong Q., Cai J.X., Zhang W.P., Sun J.Y., Wang T.T., Xie J., He H.R., Xing J.F. (2013). Establishment of paclitaxel-resistant breast cancer cell line and nude mice models, and underlying multidrug resistance mechanisms in vitro and in vivo. Asian Pac. J. Cancer Prev..

[43] Zhang J., Zhao J., Zhang W., Liu G., Yin D., Li J., Zhang S., Li H. (2012). Establishment of paclitaxel-resistant cell line and the underlying mechanism on drug resistance. Int. J. Gynecol. Cancer.

[44] Nunes M., Silva P.M.A., Coelho R., Pinto C., Resende A., Bousbaa H., Almeida G.M., Ricardo S. (2021). Generation of Two Paclitaxel-Resistant High-Grade Serous Carcinoma Cell Lines with Increased Expression of P-Glycoprotein. Front. Oncol..

[45] Aldonza M.B., Hong J.Y., Lee S.K. (2017). Paclitaxel-resistant cancer cell-derived secretomes elicit ABCB1-associated docetaxel cross-resistance and escape from apoptosis through FOXO3a-driven glycolytic regulation. Exp. Mol. Med..

[46] He Y., He Z., Lin J., Chen C., Chen Y., Liu S. (2021). CtBP1/2 differentially regulate genomic stability and DNA repair pathway in high-grade serous ovarian cancer cell. Oncogenesis.

[47] Logan C.M., Menko A.S. (2019). Microtubules: Evolving roles and critical cellular interactions. Exp. Biol. Med..

[48] Orr G.A., Verdier-Pinard P., McDaid H., Horwitz S.B. (2003). Mechanisms of Taxol resistance related to microtubules. Oncogene.

[49] Saiki Y., Yoshino Y., Fujimura H., Manabe T., Kudo Y., Shimada M., Mano N., Nakano T., Lee Y., Shimizu S. (2012). DCK is frequently inactivated in acquired gemcitabine-resistant human cancer cells. Biochem. Biophys. Res. Commun..

[50] Doench J.G., Fusi N., Sullender M., Hegde M., Vaimberg E.W., Donovan K.F., Smith I., Tothova Z., Wilen C., Orchard R. (2016). Optimized sgRNA design to maximize activity and minimize off-target effects of CRISPR-Cas9. Nat. Biotechnol..

[51] Hsu P.D., Scott D.A., Weinstein J.A., Ran F.A., Konermann S., Agarwala V., Li Y., Fine E.J., Wu X., Shalem O. (2013). DNA targeting specificity of RNA-guided Cas9 nucleases. Nat. Biotechnol..

[52] Sanjana N.E., Shalem O., Zhang F. (2014). Improved vectors, and genome-wide libraries for CRISPR screening. Nat. Methods.

[53] Shalem O., Sanjana N.E., Hartenian E., Shi X., Scott D.A., Mikkelson T., Heckl D., Ebert B.L., Root D.E., Doench J.G. (2014). Genome-scale CRISPR-Cas9 knockout screening in human cells. Science.

[54] Akhtar J., Han Y., Han S., Lin W., Cao C., Ge R., Babarinde I.A., Jia Q., Yuan Y., Chen G. (2022). Bistable insulin response: The win-win solution for glycemic control. iScience.

[55] Schneider C.A., Rasband W.S., Eliceiri K.W. (2012). NIH Image to ImageJ: 25 years of image analysis. Nat. Methods.

[56] Yue P.Y., Leung E.P., Mak N.K., Wong R.N. (2010). A simplified method for quantifying cell migration/wound healing in 96-well plates. J. Biomol. Screen..

