# Supplementary material for: CRISPR/Cas9-Mediated CtBP1 Gene Editing Enhances Chemosensitivity and Inhibits Metastatic Potential in Esophageal Squamous Cell Carcinoma Cells
Source: Int J Mol Sci. 2023 Sep 13;24(18):14030. doi: 10.3390/ijms241814030 (PMC10530806; doi:10.3390/ijms241814030)
Supplement: Supplementary file 1 [file ijms-24-14030-s001.zip › ijms-2407211-supplementary.pdf]

International Journal of *Molecular Sciences*

MDPI

Article

CRISPR/Cas9-mediated CtBP1 Gene Editing Enhances Chemosensitivity and Inhibits Metastatic Potential in Esophageal Squamous Cell Carcinoma Cells

Javed Akhtar[1,2,3,4], Muhammad Imran[5] and Guanyu Wang[1,2,3,4,*]

1 Futian Biomedical Innovation R&D Center, The Chinese University of Hong Kong, Shenzhen 518172, China
2 Biomedical Science and Engineering, School of Medicine, The Chinese University of Hong Kong, Shenzhen 518172, China
3 Ciechanover Institute of Precision and Regenerative Medicine, School of Medicine, The Chinese University of Hong Kong, Shenzhen 518172, China
4 Center for Endocrinology and Metabolic Diseases, Second Affiliated Hospital, The Chinese University of Hong Kong, Shenzhen 518172, China
5 Department of Computer Science & IT, Institute of Southern Punjab, Multan, Pakistan
* Correspondence: wangguanyu@cuhk.edu.cn

Supplementary Materials

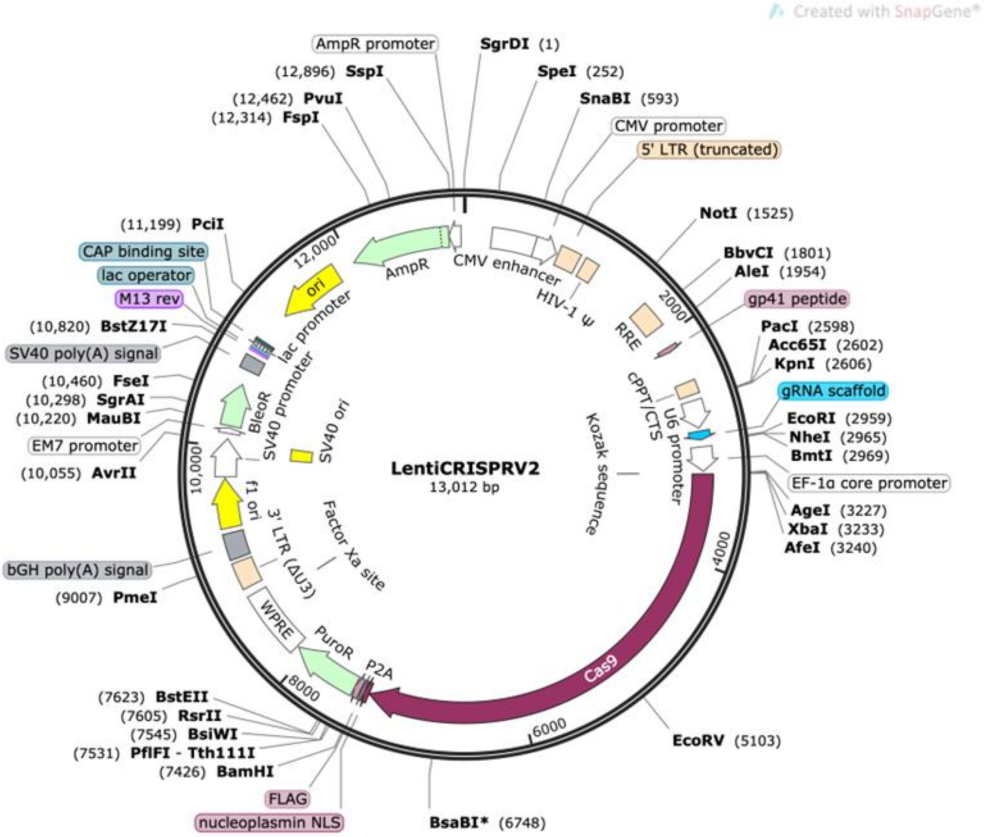

Figure S1. Map of LentiCRISPRv2. LentiCRISPRv2 (also known as pLentiCRISPR v2) is a widely used lentiviral transfer plasmid for CRISPR/Cas9 genome editing in mammalian cells with a single guide RNA (sgRNA). (The figure is adapted from https://www.addgene.org/52961/).The vector contains a constitutively active human or mouse U6 promoter to drive expression of a custom-designed guide RNA (gRNA), a Cas9 expression cassette under the control of a human or mouse EF-1α promoter, and a puromycin resistance gene for antibiotic selection of transduced cells. The vector

design also incorporates a self-cleaving 2A peptide sequence to separate the Cas9 and gRNA sequences during translation. Upon transduction, the Cas9 nuclease can be guided by the gRNA to a specific location in the genome, leading to targeted double-strand breaks that can stimulate DNA repair mechanisms and enable targeted gene knockout, insertion, or modification. The efficient and stable delivery of the Cas9 and gRNA expression cassettes via the LentiCRISPRv2 vector system makes it a valuable tool for genome editing in a wide variety of cell types and organisms.

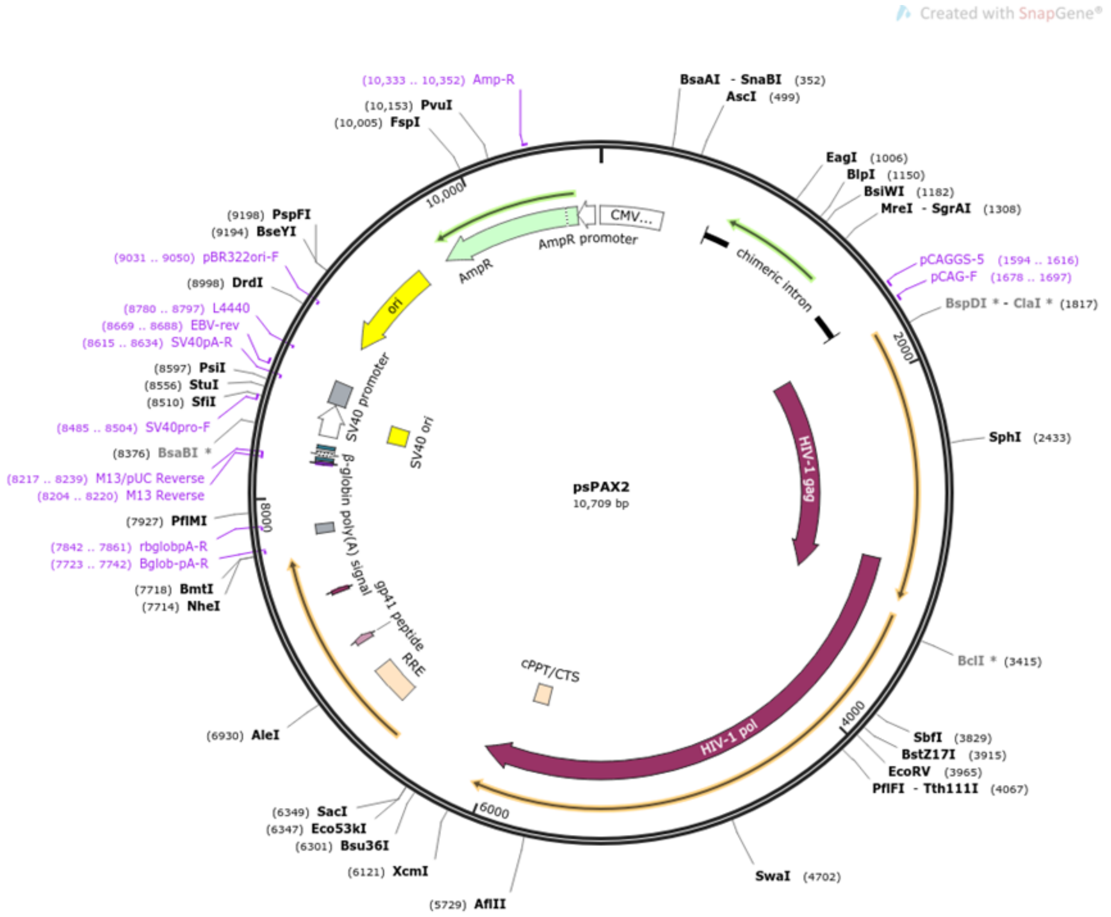

Figure S2. Map of psPAX2. psPAX2 is a second-generation lentiviral packaging plasmid widely used too to produce lentiviral vectors. (The figure is adapted from https://www.addgene.org/12260/).The plasmid contains several key elements, including a CMV promoter to drive high-level expression of the gene of interest, a packaging sequence for the lentiviral genome, and a puromycin resistance gene for selection of transduced cells. The PSPAX2 plasmid encodes the Gag, Pol, and Rev proteins required for lentivirus packaging and particle production. Co-transfection of the PSPAX2 plasmid with a lentiviral vector plasmid and an envelope plasmid enables the efficient and reliable production of high-titer lentiviral particles for gene delivery.

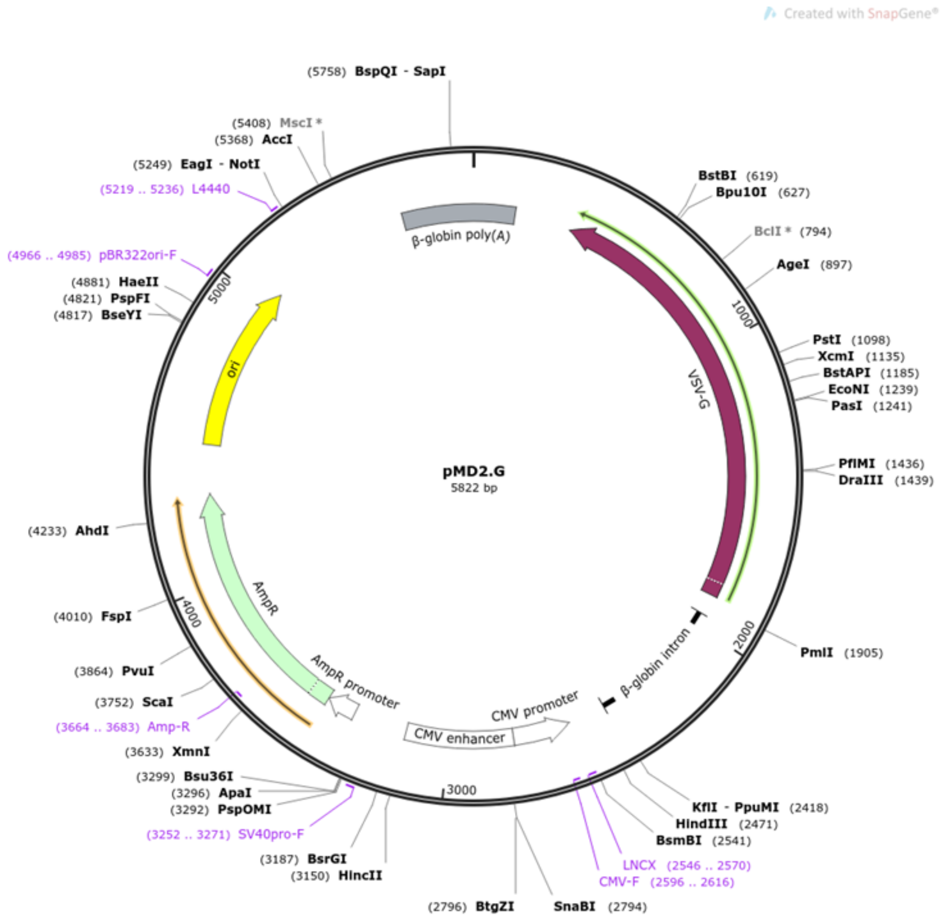

Figure S3. Map of pMD2G. pMD2G is a VSV-G envelope expressing plasmid commonly used for producing lentiviral vectors. (The figure is adapted from https://www.addgene.org/12259/). The plasmid contains several key elements, including the CMV promoter to drive high-level expression of the gene of interest, a cDNA encoding the VSV-G envelope glycoprotein, and a neomycin resistance gene for selection of transduced cells. The VSV-G envelope gly-coprotein facilitates the entry of the lentiviral vector into target cells by mediating fusion with the host cell membrane. The pMD2.G plasmid is typically co-transfected with a lentiviral vector plasmid and a packaging plasmid to produce high-titer lentiviral particles for gene delivery. pMD2.G is an efficient and reliable for production of lentiviral vector.

OFF TARGET SEQUENCES

Filters

OFF TARGET SITE	MISMATCHES	CHROMOSOME	CUT SITE	PAM	GENE
AATCACTGAAGCCTGGGAGG	3	chr19	5,738,674	CGG	CATSPERD
AATCACTGACGCCTGCCTCA	3	chr8	138,764,465	GGG	COL22A1
CATCACTGAAGCCTGTGTAG	3	chr8	143,712,987	GGG	RP11-429J17.4
AGTCACTGAAGCCTGAGTGG	3	chr10	71,069,175	TGG	
AATCACTGGAGCCTGCGAAG	3	chr19	33,170,790	CGG	WDR88
TAACACTGAAGCCTGCCTCA	4	chr1	222,984,009	AGG	RP11-455P21.3
AATCACTCAAACCTGAGTGG	4	chr1	227,962,801	TGG	
AATCACTTAAGCCTGGGAGG	4	chr11	36,708,551	TGG	
AATCACTGTAGCCTGAGGCA	4	chr10	3,193,529	GGG	
AATCACTTGAGCCTGGGACG	4	chr10	11,917,018	GGG	
[illegible]	4	[illegible]	[illegible]	[illegible]	[illegible]

Figure S4. Potential Off target sequences.

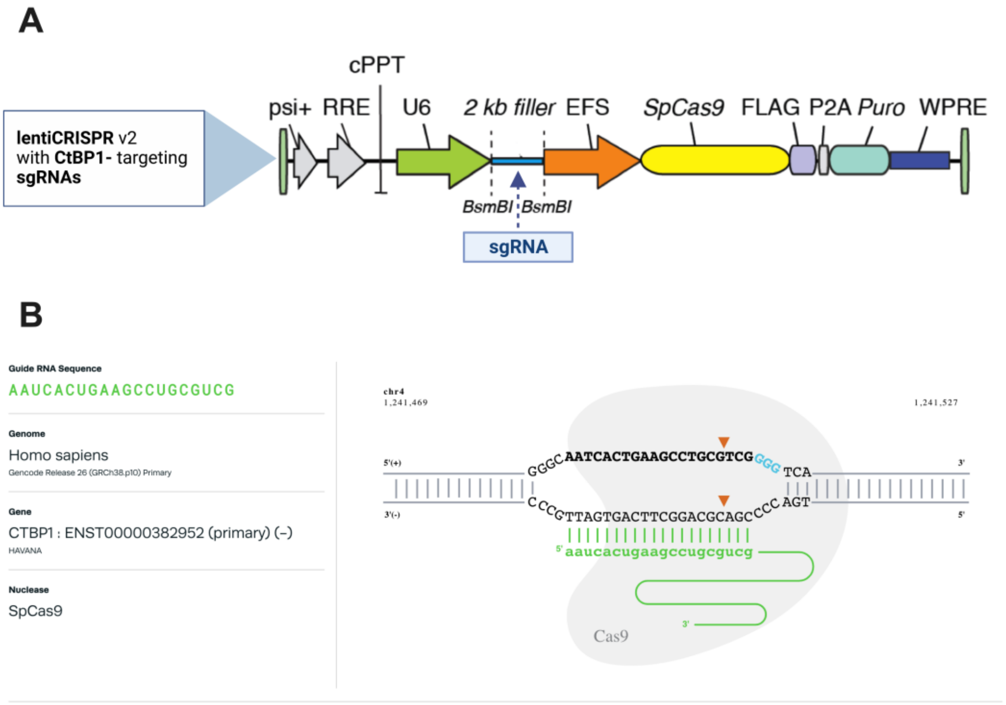

Figure S5. Efficient sgRNA Design for CtBP1 Gene Editing with CRISPR/Cas9. (A) Map of the LentiCRISPRv2 vector with a CtBP1 sgRNA. (This figure is adapted from http://genome-engineering.org/gecko/wp-content/uploads/2013/12/lentiCRISPRv2-and-lentiGuide-oligo-cloning-protocol.pdf). **(B)** Specifically, guide targets the primary transcript of the CtBP1 gene (ENST00000382952) in homo sapiens. Cas9/gRNA ribonucleoprotein (RNP) binds to the antisense strand (-) of the gene and creates a site-specific dou-ble-stranded break at position 1,241,503.
